# Supplementary material for: Understanding the influence of socioeconomic status on the association between combinations of lifestyle factors and adverse health outcomes: a systematic review protocol
Source: BMJ Open. 2021 May 27;11(5):e042212. doi: 10.1136/bmjopen-2020-042212 (PMC8162079; doi:10.1136/bmjopen-2020-042212)
Supplement: Author's manuscript [file bmjopen-2020-042212.draft_revisions.pdf]

BMJ Open is committed to open peer review. As part of this commitment we make the peer review history of every article we publish publicly available.

When an article is published we post the peer reviewers' comments and the authors' responses online. We also post the versions of the paper that were used during peer review. These are the versions that the peer review comments apply to.

The versions of the paper that follow are the versions that were submitted during the peer review process. They are not the versions of record or the final published versions. They should not be cited or distributed as the published version of this manuscript.

BMJ Open is an open access journal and the full, final, typeset and author-corrected version of record of the manuscript is available on our site with no access controls, subscription charges or pay-per-view fees (http://bmjopen.bmj.com).

If you have any questions on BMJ Open's open peer review process please email info.bmjopen@bmj.com

BMJ Open

# Understanding the influence of socioeconomic status on the association between combinations of lifestyle factors and adverse health outcomes: a systematic review protocol.



| | |
|---|---|
| Journal: | *BMJ Open* |
| Manuscript ID | bmjopen-2020-042212 |
| Article Type: | Protocol |
| Date Submitted by the Author: | 28-Jun-2020 |



| | |
|---|---|
| Complete List of Authors: | Foster, Hamish; University of Glasgow, General Practice and Primary Care; University of Glasgow<br>Polz, Peter; University of Glasgow, General Practice and Primary Care<br>Mair, Frances; University of Glasgow, General Practice and Primary Care<br>Gill, Jason; University of Glasgow, Institute of Cardiovascular and Medical Sciences<br>O'Donnell, Catherine; University of Glasgow, General Practice & Primary Care |



| | |
|---|---|
| Keywords: | EPIDEMIOLOGY, PREVENTIVE MEDICINE, PUBLIC HEALTH, SOCIAL MEDICINE |


SCHOLARONE™
Manuscripts

*I, the Submitting Author has the right to grant and does grant on behalf of all authors of the Work (as defined in the below author licence), an exclusive licence and/or a non-exclusive licence for contributions from authors who are: i) UK Crown employees; ii) where BMJ has agreed a CC-BY licence shall apply, and/or iii) in accordance with the terms applicable for US Federal Government officers or employees acting as part of their official duties; on a worldwide, perpetual, irrevocable, royalty-free basis to BMJ Publishing Group Ltd ("BMJ") its licensees and where the relevant Journal is co-owned by BMJ to the co-owners of the Journal, to publish the Work in this journal and any other BMJ products and to exploit all rights, as set out in our licence.*

*The Submitting Author accepts and understands that any supply made under these terms is made by BMJ to the Submitting Author unless you are acting as an employee on behalf of your employer or a postgraduate student of an affiliated institution which is paying any applicable article publishing charge ("APC") for Open Access articles. Where the Submitting Author wishes to make the Work available on an Open Access basis (and intends to pay the relevant APC), the terms of reuse of such Open Access shall be governed by a Creative Commons licence – details of these licences and which Creative Commons licence will apply to this Work are set out in our licence referred to above.*

*Other than as permitted in any relevant BMJ Author's Self Archiving Policies, I confirm this Work has not been accepted for publication elsewhere, is not being considered for publication elsewhere and does not duplicate material already published. I confirm all authors consent to publication of this Work and authorise the granting of this licence.*

For peer review only

# Understanding the influence of socioeconomic status on the association between combinations of lifestyle factors and adverse health outcomes: a systematic review protocol.

Hamish M.E. Foster[1], Peter Polz[1], Frances S. Mair[1], Jason M.R. Gill[2], and Catherine A. O'Donnell[1]

1. General Practice and Primary Care, Institute of Health and Wellbeing, College of Medical, Veterinary and Life Sciences, University of Glasgow, Glasgow, UK
2. Institute of Cardiovascular and Medical Sciences, College of Medical, Veterinary and Life Sciences, University of Glasgow, Glasgow, UK

Corresponding author:

Prof. Catherine A. O'Donnell

General Practice and Primary Care, University of Glasgow

1 Horselethill Road, Glasgow, G12 9LX, UK

Email: Kate.O'Donnell@glasgow.ac.uk



For peer review only - http://bmjopen.bmj.com/site/about/guidelines.xhtml

Tel: 0141 330 8329

For peer review only



For peer review only - http://bmjopen.bmj.com/site/about/guidelines.xhtml

# ABSTRACT

## Introduction

Combinations of unhealthy lifestyle factors are strongly associated with mortality, cardiovascular disease (CVD) and cancer. It is unclear how socioeconomic status (SES) affects those associations. This systematic review aims to synthesise studies that examine how SES moderates the association between lifestyle factor combinations and adverse health outcomes.

## Methods and analysis

Three databases will be searched (PubMed, EMBASE, CINAHL) from inception to March 2020. Reference lists, citations, and grey literature will also be searched. Inclusion criteria are: 1) prospective cohort studies; 2) investigations of two key exposures- i) lifestyle factor combinations of at least three lifestyle factors (e.g. smoking, physical activity, and diet); and ii) SES (e.g. income, education, or poverty index); 3) an assessment of the impact of SES on the association between combinations of unhealthy lifestyle factors and health outcomes; 4) at least one outcome from - mortality (all-cause, CVD, and cancer), CVD or cancer incidence. Two independent reviewers will screen titles, abstracts and full texts of included studies. Data extraction will focus on cohort characteristics, exposures, direction and magnitude of SES effects, methods, and quality (assessed via Newcastle-Ottawa Scale). If appropriate, a meta-analysis, pooling the effects of SES, will be performed. Alternatively, a synthesis without meta-analysis will be conducted.



For peer review only - http://bmjopen.bmj.com/site/about/guidelines.xhtml

## Ethics and dissemination

This systematic review will be the first to synthesise the evidence for whether and how SES moderates the association between combinations of unhealthy lifestyle factors and adverse health outcomes. Greater understanding of how lifestyle risk varies across socioeconomic spectra could help prevent adverse health outcomes by 1) identifying novel high-risk groups or targets for future interventions; and 2) informing research, policy, and public health interventions that aim to support healthy lifestyles in socioeconomically deprived communities. Ethical approval is not required as this review will not involve individual-level data. Results will be disseminated via peer-reviewed publication, professional networks, social media, public events and conference presentations.

## Study registration number: PROSPERO CRD42020172588

## Keywords

Healthcare disparities; Lifestyle; Mortality; Cardiovascular diseases; Neoplasms

## Strengths and limitations of this study

- This systematic review will be the first to synthesise the current evidence for the effect of socioeconomic factors on the association between combinations of unhealthy lifestyle factors and adverse health outcomes.
- Study design and methodology of included studies are likely to vary significantly and therefore it may be necessary to conduct a synthesis without meta-analysis.

For peer review only

- This review will consolidate and deepen understanding of the interactions between socioeconomic conditions and lifestyle that contribute to adverse health outcomes.

For peer review only



For peer review only - http://bmjopen.bmj.com/site/about/guidelines.xhtml

# INTRODUCTION

## Background

Globally, unhealthy lifestyle factors (e.g. smoking, physical inactivity) are among the main risk factors for mortality and non-communicable diseases (NCDs).(1) Socioeconomically deprived populations have the highest mortality and morbidity rates from NCDs but this is only partially explained by higher prevalence of unhealthy lifestyle factors.(2-4) Deeper explanations for lifestyle-related health inequalities include both the synergistic interactions between individual lifestyle factors themselves and interactions between lifestyle factors and socioeconomic status (SES).(5, 6) However, to date, there has been limited examination of how the association between combinations of unhealthy lifestyle factors and adverse health outcomes is moderated by SES.

For peer review only

## Combinations of Lifestyle Factors

While single lifestyle factors are clearly associated with adverse health outcomes, meta-analyses provide evidence for how combinations of lifestyle factors have stronger associations with mortality and NCDs.(5, 7, 8) The evidence for the health impact of single lifestyle factors now also includes ‘new’ or emerging lifestyle factors, such as sleep duration,(9) television viewing time,(10) and social participation levels.(11) When ‘new’ lifestyle factors are combined with ‘conventional’ factors (e.g. smoking, physical inactivity, high alcohol intake, or poor diet) associations with mortality are strengthened further.(11) Moreover, combinations of lifestyle factors can have a multiplicative or synergistic effect on adverse health outcomes. For example, the mortality associated with smoking and high



For peer review only - http://bmjopen.bmj.com/site/about/guidelines.xhtml

alcohol intake together is more than the sum of the risks for each lifestyle factor alone.(12) Therefore, investigating the impact of broad combinations of lifestyle factors is necessary for comprehensive understanding of lifestyle-associated harm. Particularly so when the prevalence of three or more lifestyle risk factors is high.(13) For example, 55% of the Scottish population has three or more risk factors.(14)

## Socioeconomic Status and Lifestyle

There is a significant body of research that focusses on investigating the extent to which the greater prevalence and nature of unhealthy lifestyle factors in lower SES populations can explain the well-known socioeconomic gradient in adverse health – so called 'differential exposure'.(3, 4, 15-20) These studies estimate that 30-50% of socioeconomic inequalities in all-cause and cause-specific mortality are attributable to the differential exposure to unhealthy lifestyle factors. Typically, these studies examine conventional lifestyle factors only, albeit often alongside metabolic factors such as blood pressure or body mass index (BMI).

However, socioeconomic factors themselves exert an independent effect on health and SES should be considered a risk factor alongside lifestyle factors.(21) Nonetheless, studies frequently lack an assessment of the interaction between unhealthy lifestyle factors and SES. There is some evidence for interactions between single lifestyle factors and SES, whereby, for the same level of exposure, lifestyle factors have different effects across socioeconomic spectra – i.e. 'differential vulnerability'.(20) For example, in a Scottish cohort, lower SES (measured by education level, social class, household income, and area-based deprivation) had stronger associations with alcohol-related hospital admissions and alcohol related-deaths



For peer review only - http://bmjopen.bmj.com/site/about/guidelines.xhtml

For peer review only

at the same level of alcohol intake even after controlling for drinking patterns, smoking and BMI.(22) Similarly, excess harm in lower SES groups has been associated with the single lifestyle factors of smoking and physical inactivity.(23)

More recently, there has been investigation of the interaction between combinations of lifestyle factors and SES.(3, 6, 20, 24, 25) However, to our knowledge, there has been no systematic review of the evidence for interactions between SES and combinations of unhealthy lifestyle factors and the effect on health. This paper describes the protocol for a systematic review of the effects of SES on the association between combinations of unhealthy lifestyle factors and adverse health outcomes. This review will highlight evidence gaps and deepen our understanding of the complex interplay between lifestyle, SES, and adverse health outcomes. Findings from this review will inform the development of policy and research that aims to better support and understand healthy lifestyles and contribute to reducing the excess lifestyle-related mortality and morbidity in lower SES populations.

## Aims

This review aims to identify, appraise and synthesise the findings from studies that examine the effects of SES on the association between combinations of unhealthy lifestyle factors and adverse health outcomes. This review has 2 key questions:

1) What are the characteristics of studies that examine the effect of SES on the association between combinations if unhealthy lifestyle factors and adverse health outcomes?
2) What is the evidence for whether and how the association between combinations if unhealthy lifestyle factors and adverse health outcomes is moderated by SES?



For peer review only - http://bmjopen.bmj.com/site/about/guidelines.xhtml

## Methods and Analysis

This systematic review is registered with the international database of prospectively registered systematic reviews (PROSPERO CRD42020172588).(26)

### Eligibility Criteria

Inclusion criteria are presented in Table 1 according to an adapted PICOS (population intervention, comparator, outcome, study design) framework from the Cochrane Handbook, where 'I' (intervention) is replaced with 'E' (exposure).(27)

Table 1. PICOS inclusion and exclusion criteria

| **PICOS element** | **Description** |
|---|---|
| Population | General population, with no restrictions on age, sex, or other sociodemographic characteristics. Cohort studies focussing on participants with an index condition/disease will be excluded. |
| Exposure | Studies that examine two main exposures of interest will be included:<br><br>1) **Lifestyle factor combinations**<br>Combinations must include at least three lifestyle factors and may include any combination of either conventional or emerging lifestyle factors. Combinations may include metabolic or intermediate risk factors (e.g. blood pressure, cholesterol, or body mass index) but at least three factors included in the combination must be behavioural lifestyle factors (e.g. smoking, physical activity, and diet) as opposed to intermediate or metabolic factors.<br><br>2) **Socioeconomic status**<br>All SES measures will be permitted. Anticipated variables include but are not limited to individual or area-based measures of education, employment, occupation, income, and deprivation or poverty indices. |
| Comparator | Studies will be included where reported findings allow an assessment of the impact of SES on the association between combinations of lifestyle factors and adverse health outcomes. |



For peer review only - http://bmjopen.bmj.com/site/about/guidelines.xhtml

| | |
|---|---|
| | Comparisons of effects for available outcomes will be made e.g. hazard ratios (HRs) of participants with the 'unhealthiest' lifestyle factor combination in the most affluent SES group will be compared with the HRs of participants with the unhealthiest lifestyle but in the least affluent SES group (i.e. unhealthy + high SES vs. unhealthy + low SES).<br><br>We will compare results for tests of interaction between lifestyle combinations and SES measures. |
| Outcomes | Primary outcome:<br>• All-cause mortality<br><br>Secondary outcomes:<br>• CVD and cancer mortality<br>• CVD and cancer incidence |
| Study | Prospective observational cohort studies.<br>Studies published in English language. |
| Exclusions | Ineligible publication/study design (e.g. reviews, conference abstracts, case control and cross-sectional studies, intervention studies, qualitative studies).<br><br>Studies lacking exposures or outcomes of interest (e.g. combinations of fewer than three lifestyle factor or SES not examined).<br><br>Studies that do not provide an assessment of the impact of SES on the association between combinations of lifestyle factors and adverse health. |

SES = socioeconomic status, CVD = cardiovascular disease.

## Population

This review will focus on the impact of SES on lifestyle associated adverse health outcomes in the general population. Of the studies included in previous systematic reviews investigating the adverse health outcomes associated with combinations of lifestyle factors, very few included an evaluation of the impact of SES.(5, 7, 28) Therefore, because it was anticipated that few studies would fit the inclusion criteria, the population type was not restricted in order to identify as many studies as possible.

For peer review only

## Exposure

Only studies that examine both combinations of lifestyle factors and SES as exposure variables will be included. Studies that examine the combined influence of at least three lifestyle factors will be included. It was decided that three lifestyle factors represented a balance between identifying the evidence for combinations of lifestyle factors as opposed to single lifestyle factors (two lifestyle factors was felt to be too narrow) whilst ensuring that a sufficient number of studies are included (there are fewer studies examining the risks of >3 lifestyle factors). In order to help identify as much literature as possible we decided that all definitions of SES variables will be accepted.

## Comparator

Studies will be included if they examine the effect of SES on the associations between combinations of lifestyle factors and adverse health outcomes. Results for effects may be reported in different ways: hazard ratios, odds ratios, incidence rates. Where possible, comparisons of effects for similar outcomes will be made across studies. For example, the hazard ratios (HRs) of participants with the least healthy lifestyle factor combination in the most affluent SES group will be compared with the HRs of participants with the least healthy lifestyle combination but in the least affluent SES group. Where reported, we will compare results for tests of interaction between lifestyle combinations and SES measures.

## Outcomes

Our primary outcome of interest is all-cause mortality. However, lifestyle-associated adverse health is well recognised to be strongly linked to CVD and cancer outcomes. Therefore, we will include studies that examine the following outcomes: CVD and cancer mortality; CVD and



For peer review only - http://bmjopen.bmj.com/site/about/guidelines.xhtml

cancer incidence. Studies examining specific CVD or cancer outcomes such as stroke, angina, or site-specific cancer will also be included. The International Classification of Diseases ($10^{th}$ revision) codes I05–I89.9 and C00-C97 will be used to define CVD and cancer outcomes, respectively.

## Study Design

We aim to identify prospective observational cohort studies. Case-control and cross-sectional studies, intervention studies, qualitative work, and review articles will be excluded. Only full-text published articles will be included and conference abstracts, dissertations, editorials, or papers without data will be excluded. Studies not published in the English language will be excluded.

## Study Identification

### Electronic Searches

A systematic search of PubMed, EMBASE and CINAHL databases will be performed. The search strategy will incorporate a combination of Medical Subject Heading (MeSH) terms and keywords. The search strategy of a recent systematic review examining combined lifestyle factors and the risk of incident type 2 diabetes was used as a template and adapted to incorporate SES related MeSH terms and keywords.(7) The search strategy has been developed with assistance from a specialist university librarian. Table 2 shows the search strategy that will be used for PubMed. The search strategy will be adapted and applied to other databases and will be available from PROSPERO once the review is complete.

For peer review only



For peer review only - http://bmjopen.bmj.com/site/about/guidelines.xhtml

## Table 2. PubMed search strategy

| | MeSH Terms and keywords | Theme |
|---|---|---|
| #1 | combination*[tiab] OR combined[tiab] OR composite[tiab] OR integrated[tiab] OR interaction*[tiab] OR joint effect* OR merged effect*[tiab] OR score*[tiab] OR adhere* to[tiab] OR collective[tiab] OR cumulative[tiab] OR multiple[tiab] | combined |
| #2 | life style[MeSH] OR life style*[tiab] OR lifestyle*[tiab] OR risk reduction behavior[MeSH] OR risk reduction behaviour*[tiab] OR health behavior[MeSH] OR health behaviour[tiab] OR health factor*[tiab] OR low risk*[tiab] OR prevention guideline*[tiab] OR protective factor*[tiab] OR risk reduction behaviour*[tiab] OR health* behaviour*[tiab] OR risk behaviour*[tiab] OR modifiable factors[tiab] | lifestyle factors |
| #3 | healthcare disparities[MeSH] OR healthcare disparities[tiab] OR Health Status Disparities[MeSH] OR disparate[tiab] OR disparit*[tiab] OR inequal*[tiab] OR health inequalities[tiab] OR unequal[tiab] OR health inequities[tiab] OR inequit*[tiab] OR socioeconomic factors[MeSH] OR socioeconomic factors[tiab] OR socio-economic*[tiab] OR socioeconomic*[tiab] OR social-economic[tiab] OR Social Determinants of Health[MeSH] OR social determinant*[tiab] OR poverty[MeSH] OR poverty[tiab] OR depriv*[tiab] OR sociological factors[MeSH] OR sociological factors[tiab] OR social medicine[MeSH] OR social medicine[tiab] | SES |
| #4 | cohort studies[MeSH] OR cohort[tiab] OR incidence[MeSH] OR incidence[tiab] OR survival analysis[MeSH] OR survival[tiab] OR early diagnosis[MeSH] OR early diagnosis[tiab] OR prospective*[tiab] OR follow* up[tiab] OR longitudinal[tiab] OR nested case-control[tiab] OR nested case control[tiab] OR predict*[tiab] | study design |
| #5 | #1 AND #2 AND #3 AND #4 | - |
| #6 | Death[MeSH] OR death*[tiab] OR mortality[MeSH] OR mortalit*[tiab] OR fatal*[tiab] OR life expectanc*[tiab] OR surviv*[tiab] | mortality outcome |
| #7 | cardiovascular diseases[MeSH] OR cardiovascular[tiab] OR CVD[tiab] OR heart disease*[tiab] OR myocardial ischaemia[tiab] OR AMI[tiab] OR IHD[tiab] OR CHD[tiab] OR coronary artery disease*[tiab] OR CAD[tiab] OR myocardial infarction[tiab] OR heart infarction[tiab] OR acute coronary syndrome[tiab] OR ACS[tiab] OR heart failure[tiab] OR sudden cardiac death[tiab] OR cerebrovascular disorder*[tiab] OR cerebrovascular accident*[tiab] OR cerebrovascular attack*[tiab] OR CVA[tiab] OR cerebrovascular disease*[tiab] OR CBVD[tiab] OR cerebral arterial disease*[tiab] OR stroke*[tiab] OR apoplex*[tiab] | CVD outcomes |
| #8 | neoplasms[MeSH] OR neoplas*[tiab] OR cancer*[tiab] OR carcinoma*[tiab] OR tumour*[tiab] OR malignanc*[tiab] | cancer outcomes |
| #9 | #6 OR #7 OR #8 | - |
| #10 | #9 AND #5 | - |

*[MeSH] = Medical Subject Heading; [tiab] = contained in either title or abstract; underlined = both UK and American spellings will be searched; *= any group of letters/characters, including no character.*

Searches will be from database inception to March 2020. Searches will be supplemented by hand searching of reference lists of included papers, forward citation searching and a search for grey literature using the following sources:

- Charities/Health organisations: e.g. The King's fund, The Health Foundation, Cancer Research UK, World Health Organisation, American Heart Association, American Cancer Society



For peer review only - http://bmjopen.bmj.com/site/about/guidelines.xhtml

- Databases such as OpenGrey, the Healthcare Management Information Consortium, the National Technical Information Service
- Google and Google Scholar
- Literature compiled by governmental organisations e.g. Department of Health in England, Office for National Statistics, Centers for Disease Control and Prevention

## Data Collection and Analysis

### Study Selection

Studies identified by the search strategy will be uploaded to 'DistillerSR' software and duplicates will be removed. Two reviewers will independently screen titles and abstracts using the inclusion criteria above. Any conflicts shall be resolved by discussion and if an agreement cannot be made the study shall be included for full-text screening.

Full-texts will be reviewed using a piloted checklist based on the inclusion and exclusion criteria. Conflicts will be resolved by discussion and will include a third reviewer if no consensus is reached. All studies excluded at the full-text stage will be listed with reasons for exclusion given.

### Data Extraction

Each study that meets inclusion criteria after full-text screening will go through the data extraction phase. Data extraction will be carried out by two reviewers working independently using a piloted data extraction form (Box 1). Data will be extracted for the following study

For peer review only

characteristics: author, publication year, title, study cohort, number of participants, proportion female, mean age, ethnicity, setting, country, date of recruitment, duration/follow up. Details of the lifestyle or metabolic factors and SES measures used as exposure variables will be extracted. Where possible, this detail will include how and when exposure variables were measured or assessed. Data will be extracted for any included metabolic factors such as BMI, blood pressure, or cholesterol levels. Details of confounder variables and the level of missing data will be extracted. Health outcome definitions and ascertainment will be recorded. The type of analysis, statistical methodology, and confounder adjustment will be extracted. Study results, the nature of adverse health outcome associations identified, and the effect sizes that measure the impact of SES on lifestyle associated outcomes will be recorded. Whether and how sensitivity analyses were conducted will be noted. Techniques for dealing with missing data will be recorded. Studies' overall conclusions will be extracted.

For peer review only



For peer review only - http://bmjopen.bmj.com/site/about/guidelines.xhtml

## Box 1. Data extraction

### Article Identifiers

- Author
- Publication year
- Title
- Journal, Vol, Issue, Page numbers
- Source (e.g. Database, Grey Literature source, handsearching of references etc.)
- Study funding

### Study characteristics

- Study aims and objectives
- Cohort name
- Number of participants
- Proportion female
- Mean age (SD)
- Ethnicity
- Country
- Setting (e.g. general population, occupational cohort etc.)
- Study duration/follow up
- Study start and end dates

### Exposures, confounders, and outcomes

- Lifestyle and/or metabolic factors (definition; when and how measured/assessed)
- SES measures (definition; when and how measured/assessed)
- SES measures (definition; when and how measured/assessed)
- Confounder variables or covariates included
- Outcome definition
- Outcome ascertainment
- Number of participants with missing data

### Analysis characteristics

- Type of analysis (statistical methods)
- Sensitivity analysis conducted
- Methods to deal with missing data

### Results, conclusions, and quality

- Effect of SES on lifestyle-associated adverse health: yes or no
- Size of effect
- Study conclusions
- NOS score and justification

## Quality

Included studies will be assessed for quality using an adapted version of the Newcastle-Ottawa Scale for cohort studies (NOS),(29) a tool that has been used extensively for the appraisal of observational studies as described here. This scale, after piloting, has been adapted to include an assessment of confounder adjustment, sensitivity analyses, and dealing with missing data (Box 2).



For peer review only - http://bmjopen.bmj.com/site/about/guidelines.xhtml

## Box 2. Adapted Newcastle-Ottawa Scale †

### Selection

1) Representativeness of the cohort
   - a) truly representative of those about whom the study makes conclusions (adult/general/low income population) *
   - b) somewhat representative *
   - c) selected group of users e.g. nurses, volunteers
   - d) no description of the derivation of the cohort
2) Ascertainment of exposures (lifestyle factors and SES measures)
   - a) secure record (e.g. health care records) *
   - b) structured interview *
   - c) written self-report
   - d) no description
3) Analysis rigour
   - a) impact of >1 socioeconomic measure assessed*
   - b) impact of 1 socioeconomic measure assessed
4) Demonstration that participants were disease free at start of study
   - a) yes/sensitivity analysis excluding those with prior disease *
   - b) no

### Comparability (2 stars available)

1) Comparability of exposed vs unexposed on the basis of the design or analysis
   - a) analysis includes adjustment for key variables and maximises the use of available data*
   - b) study offers reasonable justification for adjustment variables *

### Outcome

1) Assessment of outcome
   - a) independent blind assessment *
   - b) record linkage *
   - c) self-report
   - d) no description
2) Was follow-up long enough for outcomes to occur
   - a) yes (≥6months) *
   - b) no
3) Adequacy of follow up of cohorts
   - a) all participants accounted for and missingness <50%, or assessment of impact of missingness given, or description of those with missing data allows assessment of impact of missingness*
   - b) some participants unaccounted for or missingness >50%, or no assessment of [illegible]



For peer review only - http://bmjopen.bmj.com/site/about/guidelines.xhtml

†A study can be awarded a maximum of one star for each numbered item within the Selection and Outcome categories. A maximum of two stars can be given for Comparability. A total of 9 stars are available: selection (4 stars), comparability (2 stars), and outcome (3 stars). If a single study combines/harmonises multiple cohorts for analysis, then the item below that best fits with the majority of the cohorts included should be given for that study. As per original NOS, for Selection, criterion 4) Demonstration that participants were disease free at start of study: 'In the case of mortality studies, outcome of interest is still the presence of a disease/ incident, rather than death. That is to say that a statement of no history of disease or incident earns a star.'(29)

## Data Synthesis

The process and results of study identification and selection based on inclusion and exclusion criteria will be displayed as a Preferred Reporting Items for Systematic Reviews and Meta-Analyses (PRISMA) flow diagram.(30)

To aid comparisons across studies we will present at least the following summary data for each included study in tabular format:

- Cohort characteristics (e.g. number of participants, proportion female, mean age, setting, date of recruitment, length of follow up)
- Lifestyle or metabolic factors included in combination
- SES measures used in assessment of SES effects
- Health outcomes and outcome ascertainment
- Risks for health outcomes and their statistical significance
- Inconsistent findings within each study
- Study quality



For peer review only - http://bmjopen.bmj.com/site/about/guidelines.xhtml

Studies will be grouped together according to our outcomes of interest (all-cause mortality; CVD and cancer mortality; CVD and cancer incidence). For all outcomes, we will describe details of how the outcome was assessed (e.g. administrative data or questionnaires) and approach used for analysis (e.g. time-to-event). We will provide details of the association between SES, lifestyle and the outcome (e.g. odds ratio, hazard ratio, etc) including the length of follow up. Where possible, comparisons of effects for similar outcomes will be made across studies. For example, we will compare HRs of participants in the least healthy category and most affluent SES category (reference group) with HRs of participants in the least healthy category but in the most deprived SES category.

If studies are sufficiently homogenous in terms of participant characteristics, exposures, and outcomes, we will standardise study findings for similar outcomes, provide justification for our transformation methods, and combine results by a random-effects meta-analysis.(31) We will then calculate $I^2$ to describe the proportion of effect estimate variance due to study heterogeneity rather than chance.

Initial scoping of the literature has identified significant exposure, outcome, and methodological heterogeneity across studies. Therefore, a synthesis without meta-analysis (SWiM) will likely be the most appropriate method to synthesise study findings.(32) As per SWiM, we will provide justifications for the method and presentation of study findings.

Irrespective of whether a meta-analysis is conducted we will provide a transparent and full account of any limitations of our synthesis. Further, in conducting and reporting this



For peer review only - http://bmjopen.bmj.com/site/about/guidelines.xhtml

systematic review we will endeavour to fulfil, where possible, all items proposed by the Meta-analysis Of Observational Studies in Epidemiology (MOOSE) Group.(33) Any amendments to the review protocol will be identified and justified upon completion.

## Patient and Public Involvement

This Systematic Review constitutes a primary aspect of HF's doctoral thesis. NHS Research Scotland Primary Care Patient and Public Involvement (NRS PPI) Group was consulted twice as part of preparatory work for the doctoral thesis funding application.(34) The NRS PPI Group were not involved in the design of the study but have influenced how results of this review, as well as other aspects of the doctoral thesis, will be presented at two planned public engagement events over the course of the thesis.

## Ethics, dissemination

This review will not require ethical approval as it will not involve individual-level patient data. Results will be disseminated via peer-reviewed publication, professional networks, social media, public events and conference presentations.

## Discussion

Both combinations of multiple unhealthy lifestyle factors and SES play major roles in mortality, CVD, and cancer.(1, 5, 7, 8, 21) Numerous studies have investigated the mediating influence of lifestyle factors in attempts to explain the socioeconomic gradient in adverse health outcomes.(3, 4) However, fewer studies appear to examine the relationships between lifestyle, SES, and adverse health outcomes from the perspective of interactions between



For peer review only - http://bmjopen.bmj.com/site/about/guidelines.xhtml

combinations of lifestyle factors and SES.(24) Understanding the evidence for whether and how SES influences the association between lifestyle and adverse health outcomes could inform policies and interventions that aim to support healthy lifestyles.

Scoping the literature suggests that evidence for a moderating influence of SES is mixed. Eguchi et al., with data from 42,647 Japanese adults aged 40–79 years and approximately 20 years follow-up, examined the risks associated with a lifestyle score (comprised of eight lifestyle factors: smoking, alcohol, physical activity, sleep, dietary intake of fruit, fish and milk, and BMI) stratified by age (≥16 or <16 years) at last formal education.(24) The authors reported a 44% higher all-cause mortality risk for those with a higher level of education but with the least healthy lifestyle, compared with those with a higher level of education but with the healthiest lifestyle. When the same comparison was made in those with the lower level of education, participants with the least healthy lifestyle had a 40% higher all-cause mortality risk compared with those with the healthiest lifestyle. Namely, the level of elevated risk associated with the least healthy lifestyle was similar in both higher and lower education groups.

Foster et al., performed similar analyses with data from 328,594 UK adults 40–69 years and approximately 5 years follow-up to examine the risks associated with a lifestyle score (comprised of nine lifestyle factors: smoking, alcohol, physical activity, television viewing time, sleep duration, and dietary intake of fruit/vegetables, oily fish, and red and processed meat) stratified by quintiles of socioeconomic deprivation (Townsend index).(6) The authors observed a 65% higher all-cause mortality risk for the least deprived with the least healthy lifestyle, compared to the least deprived but with the healthiest lifestyle. However, when the



For peer review only - http://bmjopen.bmj.com/site/about/guidelines.xhtml

same comparison was made in the most deprived participants, those with the least healthy lifestyle had a 145% higher risk than those with the healthiest lifestyles.

These highly comparable studies report opposing results. Eguchi et al. found no interaction between lifestyle and SES with similar lifestyle risks in the least and most educated groups. Whereas Foster et al. found an interaction between lifestyle and SES with disproportionately raised lifestyle risk in the most deprived group. Several methodological differences (lifestyle and SES measures; follow-up time; population characteristics) could explain the two studies' conflicting results but additional evidence from similar studies would help to clarify whether there is any moderating influence of SES on lifestyle-associated risks. This systematic review will help unpack such associations in more detail.

This systematic review will be the first to our knowledge to synthesise the evidence for whether and how SES influences the association between combinations of lifestyle factors and adverse health. We will describe the lifestyle factors, SES measures, and adverse health outcomes that have been examined thus far. We will collate and interpret the findings considering both the type of analyses and the quality of studies to provide a comprehensive synthesis of available evidence and highlight gaps in current evidence.

We have developed a comprehensive search strategy with broad inclusion criteria in order to identify all available evidence and reduce the chance of omitting relevant studies. However, our scoping of the literature suggests that not only will there be few studies that attempt to examine this problem but also that previous studies will be widely heterogeneous both in terms of the lifestyle factor and SES variables examined and in terms of the statistical methods employed. This will likely preclude a meta-analytical synthesis of the evidence, which may be



For peer review only - http://bmjopen.bmj.com/site/about/guidelines.xhtml

a limitation of our review. However, a synthesis without meta-analysis (SWiM) will likely highlight important gaps in available evidence and direct future research in this sphere. This review will adhere to SWiM reporting guidelines and will be guided by MOOSE recommendations to improve transparency and clarity.(33, 35) All screening, data extraction, and quality assessment will be performed independently by two reviewers to improve study rigour.

This comprehensive and rigorous systematic review will improve our understanding of the complex interaction between socioeconomic status and lifestyle and has the potential to inform research, interventions and policy.

## Author Contributions

HMEF, CAO'D, FSM and JMRG were involved in study concept. HMEF, CAO'D, FSM, PP and JMRG developed the study design. HMEF, CAO'D, FSM, JMRG and PP were involved in acquisition, analysis, or interpretation of data. Drafting of the manuscript was led by HMEF and PP with supervision and support from CAO'D, FSM and JMRG. All authors were involved in critical revision of the manuscript for important intellectual content. CAO'D is the guarantor.

## Funding Statement

HF is funded by a Medical Research Council Clinical Research Training Fellowship (MRC CRTF; MR/T001585/1) and this body of work constitutes a primary aspect of HF's doctoral thesis. The funder had no role in developing this protocol.



For peer review only - http://bmjopen.bmj.com/site/about/guidelines.xhtml

## Competing Interests Statement

The authors have no conflicts of interests to declare.

## Word Count

3,833

# REFERENCES

1. Stanaway JD, Afshin A, Gakidou E, Lim SS, Abate D, Abate KH, et al. Global, regional, and national comparative risk assessment of 84 behavioural, environmental and occupational, and metabolic risks or clusters of risks for 195 countries and territories, 1990-2017: a systematic analysis for the Global Burden of Disease Study 2017. Lancet. 2018;392(10159):1923-94.
2. Meader N, King K, Moe-Byrne T, Wright K, Graham H, Petticrew M, et al. A systematic review on the clustering and co-occurrence of multiple risk behaviours. BMC Public Health. 2016;16(1).
3. Laine JE, Baltar VT, Stringini S, Gandini M, Chadeau-Hyam M, Kivimaki M, et al. Reducing socio-economic inequalities in all-cause mortality: a counterfactual mediation approach. International journal of epidemiology. 2019.
4. Marmot MG, Shipley MJ, Hemingway H, Head J, Brunner EJ. Biological and behavioural explanations of social inequalities in coronary heart disease: the Whitehall II study. Diabetologia. 2008;51(11):1980-8.
5. Loef M, Walach H. The combined effects of healthy lifestyle behaviors on all cause mortality: a systematic review and meta-analysis. Prev Med. 2012;55(3):163-70.
6. Foster HM, Celis-Morales CA, Nicholl BI, Petermann-Rocha F, Pell JP, Gill JM, et al. The effect of socioeconomic deprivation on the association between an extended measurement of unhealthy lifestyle factors and health outcomes: a prospective analysis of the UK Biobank cohort. The Lancet Public Health. 2018;3(12):e576-e85.
7. Zhang Y, Pan XF, Chen J, Xia L, Cao A, Zhang Y, et al. Combined lifestyle factors and risk of incident type 2 diabetes and prognosis among individuals with type 2 diabetes: a systematic review and meta-analysis of prospective cohort studies. Diabetologia. 2020;63(1):21-33.
8. Zhang YB, Pan XF, Chen J, Cao A, Zhang YG, Xia L, et al. Combined lifestyle factors, incident cancer, and cancer mortality: a systematic review and meta-analysis of prospective cohort studies. Br J Cancer. 2020;122(7):1085-93.
9. Jike M, Itani O, Watanabe N, Buysse DJ, Kaneita Y. Long sleep duration and health outcomes: A systematic review, meta-analysis and meta-regression. Sleep Med Rev. 2018;39:25-36.

For peer review only



For peer review only - http://bmjopen.bmj.com/site/about/guidelines.xhtml

10. Sun JW, Zhao LG, Yang Y, Ma X, Wang YY, Xiang YB. Association Between Television Viewing Time and All-Cause Mortality: A Meta-Analysis of Cohort Studies. Am J Epidemiol. 2015;182(11):908-16.
11. Krokstad S, Ding D, Grunseit AC, Sund ER, Holmen TL, Rangul V, et al. Multiple lifestyle behaviours and mortality, findings from a large population-based Norwegian cohort study - The HUNT Study. Bmc Public Health. 2017;17.
12. Verplaetse TL, McKee SA. An overview of alcohol and tobacco/nicotine interactions in the human laboratory. Am J Drug Alcohol Ab. 2017;43(2):186-96.
13. Linardakis M, Smpokos E, Papadaki A, Komninos ID, Tzanakis N, Philalithis A. Prevalence of multiple behavioral risk factors for chronic diseases in adults aged 50+, from eleven European countries - the SHARE study (2004). Prev Med. 2013;57(3):168-72.
14. Lawder R, Harding O, Stockton D, Fischbacher C, Brewster DH, Chalmers J, et al. Is the Scottish population living dangerously? Prevalence of multiple risk factors: the Scottish Health Survey 2003. Bmc Public Health. 2010;10.
15. Redondo-Bravo L, Fernandez-Alvira JM, Gorriz J, Mendiguren JM, Sanz J, Fernandez-Friera L, et al. Does Socioeconomic Status Influence the Risk of Subclinical Atherosclerosis? A Mediation Model. J Am Coll Cardiol. 2019;74(4):526-35.
16. Stringhini S, Zaninotto P, Kumari M, Kivimaki M, Lassale C, Batty GD. Socio-economic trajectories and cardiovascular disease mortality in older people: the English Longitudinal Study of Ageing. International Journal of Epidemiology. 2018;47(1):36-46.
17. Syden L, Landberg, J. The contribution of alcohol use and other lifestyle factors to socioeconomic differences in all-cause mortality in a Swedish cohort. Drug and Alcohol Review. 2017;36(5):691-700.
18. Groeniger JO, Kamphuis, C.B., Mackenbach, J.P., van Lenthe, F.J. . Repeatedly measured material and behavioral factors changed the explanation of socioeconomic inequalities in all-cause mortality. Journal of clinical epidemiology. 2017;91:137-45. Journal of Clinical Epidemiology. 2017;91:137-45.
19. Stringhini S, Sabia S, Shipley M, Brunner E, Nabi H, Kivimaki M, et al. Association of Socioeconomic Position With Health Behaviors and Mortality. Jama-J Am Med Assoc. 2010;303(12):1159-66.
20. Nordahl H, Lange T, Osler M, Diderichsen F, Andersen I, Prescott E, et al. Education and Cause-specific Mortality. 2014;25(3):389-96.
21. Stringhini S, Carmeli C, Jokela M. Socioeconomic status and the 25 x 25 risk factors as determinants of premature mortality: a multicohort study and meta-analysis of 1.7 million men and women (vol 389, pg 1229, 2017). Lancet. 2017;389(10075):1194-.
22. Katikireddi SV, Whitley E, Lewsey J, Gray L, Leyland AH. Socioeconomic status as an effect modifier of alcohol consumption and harm: analysis of linked cohort data. Lancet Public Health. 2017;2(6):E267-E76.
23. Pampel FC, Rogers RG. Socioeconomic status, smoking, and health: A test of competing theories of cumulative advantage. J Health Soc Behav. 2004;45(3):306-21.
24. Eguchi E, Iso H, Honjo K, Yatsuya H, Tamakoshi A. No modifying effect of education level on the association between lifestyle behaviors and cardiovascular mortality: the Japan Collaborative Cohort Study. Sci Rep-Uk. 2017;7.
25. Andersen SW, Zheng W, Sonderman J, Shu XO, Matthews CE, Yu DX, et al. Combined Impact of Health Behaviors on Mortality in Low-Income Americans. Am J Prev Med. 2016;51(3):344-55.



For peer review only - http://bmjopen.bmj.com/site/about/guidelines.xhtml

26. Understanding the impact of socioeconomic status on the association between combined lifestyle factors and adverse health outcomes: a systematic review. PROSPERO 2020 CRD42020172588 [Internet]. 2020 [cited 25/06/2020]. Available from: https://www.crd.york.ac.uk/prospero/display_record.php?ID=CRD42020172588.
27. McKenzie J, Brennan, SE., Ryan, RE., Thomson, HJ., Johnston, RV., Thomas, J.,. Chapter 3: Defining the criteria for including studies and how they will be grouped for the synthesis. In: Cochrane Handbook for Systematic Reviews of Interventions version 6.0. . 2019.
28. Zhang YB, Pan XF, Chen J, Cao A, Zhang YG, Xia L, et al. Combined lifestyle factors, incident cancer, and cancer mortality: a systematic review and meta-analysis of prospective cohort studies. Br J Cancer. 2020.
29. Wells GA, Shea B, O'Connell D, Peterson J, Welch V, Losos M, et al. The Newcastle-Ottawa Scale (NOS) for assessing the quality of nonrandomised studies in meta-analyses: Ottawa Hospital Research Institute [Available from: http://www.ohri.ca/programs/clinical_epidemiology/oxford.asp.
30. Moher D, Liberati A, Tetzlaff J, Altman D. Preferred reporting items for systematic reviews and meta-analyses: the PRISMA statement [Guideline Research Support, Non-US Gov't]. Bmj. 2009;339:b2535.
31. Deeks J, Higgins, JPT., Altman, DG. . Chapter 10: Analysing data and undertaking meta-analyses. In: Cochrane Handbook for Systematic Reviews of Interventions version 6.0 (updated July 2019). Cochrane, 2019. Available from www.training.cochrane.org/handbook.; 2019.
32. Campbell M, McKenzie JE, Sowden A, Katikireddi SV, Brennan SE, Ellis S, et al. Synthesis without meta-analysis (SWiM) in systematic reviews: reporting guideline. BMJ. 2020;368:l6890.
33. Stroup DF, Berlin JA, Morton SC, Olkin I, Williamson GD, Rennie D, et al. Meta-analysis of observational studies in epidemiology: a proposal for reporting. Meta-analysis Of Observational Studies in Epidemiology (MOOSE) group. JAMA. 2000;283(15):2008-12.
34. The NRS Primary Care PPI Group [Available from: https://www.nhsresearchscotland.org.uk/research-areas/primary-care/get-involved.
35. Campbell M, McKenzie JE, Sowden A, Katikireddi SV, Brennan SE, Ellis S, et al. Synthesis without meta-analysis (SWiM) in systematic reviews: reporting guideline. Bmj-Brit Med J. 2020;368.



For peer review only - http://bmjopen.bmj.com/site/about/guidelines.xhtml

**PRISMA-P (Preferred Reporting Items for Systematic review and Meta-Analysis Protocols) 2015 checklist: recommended items to address in a systematic review protocol***

| Section and topic | Item No | Checklist item | Page number |
|---|---|---|---|
| **ADMINISTRATIVE INFORMATION** | | | |
| Title: | | | |
| Identification | 1a | Identify the report as a protocol of a systematic review | |
| Update | 1b | If the protocol is for an update of a previous systematic review, identify as such | 1 |
| Registration | 2 | If registered, provide the name of the registry (such as PROSPERO) and registration number | 3 |
| Authors: | | | |
| Contact | 3a | Provide name, institutional affiliation, e-mail address of all protocol authors; provide physical mailing address of corresponding author | 1 |
| Contributions | 3b | Describe contributions of protocol authors and identify the guarantor of the review | 1 |
| Amendments | 4 | If the protocol represents an amendment of a previously completed or published protocol, identify as such and list changes; otherwise, state plan for documenting important protocol amendments | 17 |
| Support: | | | |
| Sources | 5a | Indicate sources of financial or other support for the review | 21 |
| Sponsor | 5b | Provide name for the review funder and/or sponsor | 21 |
| Role of sponsor or funder | 5c | Describe roles of funder(s), sponsor(s), and/or institution(s), if any, in developing the protocol | 21 |
| **INTRODUCTION** | | | |
| Rationale | 6 | Describe the rationale for the review in the context of what is already known | 4-6 |
| Objectives | 7 | Provide an explicit statement of the question(s) the review will address with reference to participants, interventions, comparators, and outcomes (PICO) | 6 |
| **METHODS** | | | |
| Eligibility criteria | 8 | Specify the study characteristics (such as PICO, study design, setting, time frame) and report characteristics (such as years considered, language, publication status) to be used as criteria for eligibility for the review | 7-10 |
| Information sources | 9 | Describe all intended information sources (such as electronic databases, contact with study authors, trial registers or other grey literature sources) with planned dates of coverage | 11-12 |
| Search strategy | 10 | Present draft of search strategy to be used for at least one electronic database, including planned limits, such that it could be repeated | 11-12 (Table 2) |

For peer review only

| | | | |
|---|---|---|---|
| Study records: | | | |
| Data management | 11a | Describe the mechanism(s) that will be used to manage records and data throughout the review | 12 |
| Selection process | 11b | State the process that will be used for selecting studies (such as two independent reviewers) through each phase of the review (that is, screening, eligibility and inclusion in meta-analysis) | 12-13 |
| Data collection process | 11c | Describe planned method of extracting data from reports (such as piloting forms, done independently, in duplicate), any processes for obtaining and confirming data from investigators | 13-14 |
| Data items | 12 | List and define all variables for which data will be sought (such as PICO items, funding sources), any pre-planned data assumptions and simplifications | 13-15 |
| Outcomes and prioritization | 13 | List and define all outcomes for which data will be sought, including prioritization of main and additional outcomes, with rationale | 13-14 |
| Risk of bias in individual studies | 14 | Describe anticipated methods for assessing risk of bias of individual studies, including whether this will be done at the outcome or study level, or both; state how this information will be used in data synthesis | 14 |
| Data synthesis | 15a | Describe criteria under which study data will be quantitatively synthesised | 16-17 |
| | 15b | If data are appropriate for quantitative synthesis, describe planned summary measures, methods of handling data and methods of combining data from studies, including any planned exploration of consistency (such as $I^2$, Kendall's $\tau$) | 17 |
| | 15c | Describe any proposed additional analyses (such as sensitivity or subgroup analyses, meta-regression) | N/A |
| | 15d | If quantitative synthesis is not appropriate, describe the type of summary planned | 17 |
| Meta-bias(es) | 16 | Specify any planned assessment of meta-bias(es) (such as publication bias across studies, selective reporting within studies) | N/A |
| Confidence in cumulative evidence | 17 | Describe how the strength of the body of evidence will be assessed (such as GRADE) | 17 |

*** It is strongly recommended that this checklist be read in conjunction with the PRISMA-P Explanation and Elaboration (cite when available) for important clarification on the items. Amendments to a review protocol should be tracked and dated. The copyright for PRISMA-P (including checklist) is held by the PRISMA-P Group and is distributed under a Creative Commons Attribution Licence 4.0.**

*From: Shamseer L, Moher D, Clarke M, Ghersi D, Liberati A, Petticrew M, Shekelle P, Stewart L, PRISMA-P Group. Preferred reporting items for systematic review and meta-analysis protocols (PRISMA-P) 2015: elaboration and explanation. BMJ. 2015 Jan 2;349(jan02 1):g7647.*

For peer review only

BMJ Open

# Understanding the influence of socioeconomic status on the association between combinations of lifestyle factors and adverse health outcomes: a systematic review protocol.

| | |
|---|---|
| Journal: | *BMJ Open* |
| Manuscript ID | bmjopen-2020-042212.R1 |
| Article Type: | Protocol |
| Date Submitted by the Author: | 09-Dec-2020 |
| Complete List of Authors: | Foster, Hamish; University of Glasgow, General Practice and Primary Care; University of Glasgow<br>Polz, Peter; University of Glasgow, General Practice and Primary Care<br>Mair, Frances; University of Glasgow, General Practice and Primary Care<br>Gill, Jason; University of Glasgow, Institute of Cardiovascular and Medical Sciences<br>O'Donnell, Catherine; University of Glasgow, General Practice & Primary Care |
| <b>Primary Subject Heading</b>: | Epidemiology |
| Secondary Subject Heading: | Public health |
| Keywords: | EPIDEMIOLOGY, PREVENTIVE MEDICINE, PUBLIC HEALTH, SOCIAL MEDICINE |
| | |

SCHOLARONE™
Manuscripts

*I, the Submitting Author has the right to grant and does grant on behalf of all authors of the Work (as defined in the below author licence), an exclusive licence and/or a non-exclusive licence for contributions from authors who are: i) UK Crown employees; ii) where BMJ has agreed a CC-BY licence shall apply, and/or iii) in accordance with the terms applicable for US Federal Government officers or employees acting as part of their official duties; on a worldwide, perpetual, irrevocable, royalty-free basis to BMJ Publishing Group Ltd ("BMJ") its licensees and where the relevant Journal is co-owned by BMJ to the co-owners of the Journal, to publish the Work in this journal and any other BMJ products and to exploit all rights, as set out in our licence.*

*The Submitting Author accepts and understands that any supply made under these terms is made by BMJ to the Submitting Author unless you are acting as an employee on behalf of your employer or a postgraduate student of an affiliated institution which is paying any applicable article publishing charge ("APC") for Open Access articles. Where the Submitting Author wishes to make the Work available on an Open Access basis (and intends to pay the relevant APC), the terms of reuse of such Open Access shall be governed by a Creative Commons licence – details of these licences and which Creative Commons licence will apply to this Work are set out in our licence referred to above.*

*Other than as permitted in any relevant BMJ Author's Self Archiving Policies, I confirm this Work has not been accepted for publication elsewhere, is not being considered for publication elsewhere and does not duplicate material already published. I confirm all authors consent to publication of this Work and authorise the granting of this licence.*

For peer review only

# Understanding the influence of socioeconomic status on the association between combinations of lifestyle factors and adverse health outcomes: a systematic review protocol.

Hamish M.E. Foster[1], Peter Polz[1], Frances S. Mair[1], Jason M.R. Gill[2], and Catherine A. O'Donnell[1]

1. General Practice and Primary Care, Institute of Health and Wellbeing, College of Medical, Veterinary and Life Sciences, University of Glasgow, Glasgow, UK
2. Institute of Cardiovascular and Medical Sciences, College of Medical, Veterinary and Life Sciences, University of Glasgow, Glasgow, UK

Corresponding author:

Prof. Catherine A. O'Donnell

General Practice and Primary Care, University of Glasgow

1 Horselethill Road, Glasgow, G12 9LX, UK

Email: Kate.O'Donnell@glasgow.ac.uk



For peer review only - http://bmjopen.bmj.com/site/about/guidelines.xhtml

Tel: 0141 330 8329

For peer review only



For peer review only - http://bmjopen.bmj.com/site/about/guidelines.xhtml

# ABSTRACT


## Introduction

Combinations of unhealthy lifestyle factors are strongly associated with mortality, cardiovascular disease (CVD), and cancer. It is unclear how socioeconomic status (SES) affects those associations. Lower SES groups may be disproportionately vulnerable to the effects of unhealthy lifestyle factors compared to higher SES groups via interactions with other factors associated with low SES (e.g., stress) or via accelerated biological ageing. This systematic review aims to synthesise studies that examine how SES moderates the association between lifestyle factor combinations and adverse health outcomes. Greater understanding of how lifestyle risk varies across socioeconomic spectra could reduce adverse health by 1) identifying novel high-risk groups or targets for future interventions; and 2) informing research, policy, and interventions that aim to support healthy lifestyles in socioeconomically deprived communities.

## Methods and analysis

Three databases will be searched (PubMed, EMBASE, CINAHL) from inception to March 2020. Reference lists, citations, and grey literature will also be searched. Inclusion criteria are: 1) prospective cohort studies; 2) investigations of two key exposures- i) lifestyle factor combinations of at least three lifestyle factors (e.g. smoking, physical activity, and diet); and ii) SES (e.g. income, education, or poverty index); 3) an assessment of the impact of SES on the association between combinations of unhealthy lifestyle factors and health outcomes; 4) at least one outcome from - mortality (all-cause, CVD, and cancer), CVD or cancer incidence.


For peer review only

Two independent reviewers will screen titles, abstracts and full texts of included studies. Data extraction will focus on cohort characteristics, exposures, direction and magnitude of SES effects, methods, and quality (via Newcastle-Ottawa Scale). If appropriate, a meta-analysis, pooling the effects of SES, will be performed. Alternatively, a synthesis without meta-analysis will be conducted.

## Ethics and dissemination

Ethical approval is not required. Results will be disseminated via peer-reviewed publication, professional networks, social media, and conference presentations.


## Study registration number: PROSPERO CRD42020172588

## Keywords

Healthcare disparities; Lifestyle; Mortality; Cardiovascular diseases; Neoplasms


## Strengths and limitations of this study

- This review protocol lays out a comprehensive search strategy and a rigorous data extraction and synthesis plan to collate the evidence for the effect of socioeconomic factors on the association between combinations of unhealthy lifestyle factors and adverse health outcomes.



For peer review only - http://bmjopen.bmj.com/site/about/guidelines.xhtml

- The inclusive nature of the eligibility criteria, which is necessary as there are likely to be few studies in this area, means included studies may be heterogenous in design and methodology and this may preclude meta-analysis.

- The wide range of possible socioeconomic indicators and combinations of lifestyle factors likely to be included due to the inclusion criteria may make firm conclusions difficult.
- However, the wide range of possible socioeconomic indicators and combinations of lifestyle factors likely to be included due to the inclusion criteria will also permit a comprehensive overview of both sets of exposures and is likely to highlight evidence gaps.
- Synthesising a broad evidence base to provide an overview of the potential influence of SES on associations between combinations of unhealthy lifestyle factors and adverse health outcomes could indicate which combinations of unhealthy lifestyle factors are associated with the greatest risks for lower SES groups.

For peer review only



For peer review only - http://bmjopen.bmj.com/site/about/guidelines.xhtml

# INTRODUCTION

## Background

Globally, unhealthy lifestyle factors (e.g. smoking, physical inactivity) are among the main risk factors for mortality and non-communicable diseases (NCDs).(1) Socioeconomically deprived populations have the highest mortality and morbidity rates from NCDs but this is only partially explained by higher prevalence of unhealthy lifestyle factors.(2-4) Deeper explanations for lifestyle-related health inequalities include both the synergistic interactions between individual lifestyle factors themselves and interactions between lifestyle factors and socioeconomic status (SES).(5, 6) However, to date, there has been limited examination of how the association between combinations of unhealthy lifestyle factors and adverse health outcomes is moderated by SES.

It is critical to note that the word 'lifestyle' implies choice and control over health behaviours. However, lower SES (more deprived, lower income, or less educated) groups are less likely to have as much control over 'lifestyle' or health behaviours than higher SES groups. Further, 'choosing' unhealthy health behaviours may be entirely rational given specific socioeconomic contexts.(7) However, the word lifestyle is employed here as this is widely understood in the context of potentially modifiable health behaviours.

## Combinations of Lifestyle Factors

While single lifestyle factors are clearly associated with adverse health outcomes, meta-analyses provide evidence for how combinations of lifestyle factors have stronger

For peer review only



For peer review only - http://bmjopen.bmj.com/site/about/guidelines.xhtml

associations with mortality and NCDs.(5, 8, 9) The evidence for the health impact of single lifestyle factors now also includes ‘new’ or emerging lifestyle factors, such as sleep duration,(10) television viewing time,(11) and social participation levels.(12) When ‘new’ lifestyle factors are combined with ‘conventional’ factors (e.g. smoking, physical inactivity, high alcohol intake, or poor diet) associations with mortality are strengthened further.(12) Moreover, combinations of lifestyle factors can have a multiplicative or synergistic effect on adverse health outcomes. For example, the mortality associated with smoking and high alcohol intake together is more than the sum of the risks for each lifestyle factor alone.(13) Therefore, investigating the impact of broad combinations of lifestyle factors is necessary for comprehensive understanding of lifestyle-associated harm. Particularly so when the prevalence of three or more lifestyle risk factors is high.(14) For example, 55% of the Scottish population has three or more risk factors.(15) Furthermore, the additional risks associated with combinations of unhealthy lifestyle factors would motivate work to determine which combinations have the highest risk. For example, if a combination of high sedentary time together with short sleep duration and poor diet is highlighted as particularly high risk then interventions could be targeted at this specific behavioural combination.

For peer review only

## Socioeconomic Status and Lifestyle

There is a significant body of research that focusses on investigating the extent to which the greater prevalence and nature of unhealthy lifestyle factors in lower SES populations can explain the well-known socioeconomic gradient in adverse health – so called ‘differential exposure’.(3, 4, 16-21) These studies estimate that 30-50% of socioeconomic inequalities in all-cause and cause-specific mortality are attributable to the differential exposure to unhealthy lifestyle factors. Typically, these studies examine conventional lifestyle factors



For peer review only - http://bmjopen.bmj.com/site/about/guidelines.xhtml

only, albeit often alongside metabolic factors such as blood pressure or body mass index (BMI).

However, despite being independently associated with mortality and non-communicable disease at levels commensurate with those of unhealthy lifestyle factors, socioeconomic factors are often omitted from lifestyle policy.(22) Furthermore, many studies appear to lack an assessment of the interaction between unhealthy lifestyle factors and SES. There is some evidence for interactions between single lifestyle factors and SES, whereby, for the same level of exposure, lifestyle factors have different effects across socioeconomic spectra – i.e. 'differential vulnerability'.(21) For example, in a Scottish cohort, lower (as opposed to higher) SES (measured by education level, social class, household income, and area-based deprivation) had stronger associations with alcohol-related hospital admissions and alcohol related-deaths at the same level of alcohol intake even after controlling for drinking patterns, smoking and BMI.(23) Similarly, excess harm in lower SES groups has been associated with the single lifestyle factors of smoking and physical inactivity.(24) The underlying mechanisms that explain differential vulnerability remain unclear but could include interactions between lower SES and other harmful factors associated with low SES (e.g., other unhealthy lifestyle factors, stress, reduced access to health care) or be due to accelerated biological ageing in lower SES groups due to greater cumulative life-course risks (e.g., increased frequency of adverse childhood experiences, poorer childhood health).(25, 26) However, differential vulnerability shown in these observational studies may also represent an artefact of residual confounding or could be due to lack of detail in survey or interview measurements of lifestyle factors which fails to fully capture greater intensiveness (differential exposure) of unhealthy

For peer review only



For peer review only - http://bmjopen.bmj.com/site/about/guidelines.xhtml

lifestyle factors in lower SES groups (e.g., lower SES groups who drink heavily may drink more than heavy drinkers in higher SES groups).(27)

More recently, there has been investigation of the interaction between combinations of lifestyle factors and SES.(3, 6, 21, 28, 29) Some studies show lower SES being associated with disproportionately higher CVD and all-cause mortality with combinations of unhealthy lifestyle factors.(6, 30) Examining the evidence for SES influence on adverse health associated with combinations of unhealthy lifestyle factors would help unpack the evidence for and against differential vulnerability and improve our understanding of wider lifestyle associated risks across SES spectra. However, to our knowledge, there has been no systematic review of the evidence for interactions between SES and combinations of unhealthy lifestyle factors in terms of adverse health outcomes. This paper describes the protocol for a systematic review of the effects of SES on the association between combinations of unhealthy lifestyle factors and adverse health outcomes. This review will highlight evidence gaps and deepen our understanding of the complex interplay between lifestyle, SES, and adverse health outcomes. Findings from this review will inform the development of policy and research that aims to better support and understand healthy lifestyles and contribute to reducing the excess lifestyle-related mortality and morbidity in lower SES populations.

## Aims

This review aims to identify, appraise and synthesise the findings from studies that examine the effects of SES on the association between combinations of unhealthy lifestyle factors and adverse health outcomes. This review has 2 key questions:

For peer review only



For peer review only - http://bmjopen.bmj.com/site/about/guidelines.xhtml

1) What are the characteristics of studies that examine the effect of SES on the association between combinations if unhealthy lifestyle factors and adverse health outcomes?
2) What is the evidence for whether and how the association between combinations if unhealthy lifestyle factors and adverse health outcomes is moderated by SES?

## Methods and Analysis

This systematic review is registered with the international database of prospectively registered systematic reviews (PROSPERO CRD42020172588).(31)

### Eligibility Criteria

Inclusion criteria are presented in Table 1 according to an adapted PICOS (population intervention, comparator, outcome, study design) framework from the Cochrane Handbook, where ‘I’ (intervention) is replaced with ‘E’ (exposure).(32)

Table 1. PICOS inclusion and exclusion criteria

| PICOS element | Description |
|---|---|
| Population | Studies of any general population type will be included. Eligibility will not be restricted by age, sex, or other sociodemographic characteristics. Cohort studies focussing on participants with an index condition/disease will be excluded. |
| Exposure | Studies that examine two main exposures of interest will be included:<br><br>1) **Lifestyle factor combinations**<br>Combinations must include at least three lifestyle factors and may include any combination of either conventional or emerging lifestyle factors. Combinations may include metabolic or intermediate risk factors (e.g. blood pressure, cholesterol, or body mass index) but at least three factors included in the combination must be behavioural lifestyle factors (e.g. smoking, physical |

For peer review only



For peer review only - http://bmjopen.bmj.com/site/about/guidelines.xhtml

| | |
|---|---|
| | activity, and diet) as opposed to intermediate or metabolic factors.<br><br>2) **Socioeconomic status**<br>All SES measures will be permitted. Anticipated variables include but are not limited to individual or area-based measures of education, employment, occupation, income, and deprivation or poverty indices. |
| Comparator | Studies will be included where reported findings allow an assessment of the impact of SES on the association between combinations of lifestyle factors and adverse health outcomes.<br><br>Comparisons of effects for available outcomes will be made e.g. hazard ratios (HRs) of participants with the 'unhealthiest' lifestyle factor combination in the most affluent SES group will be compared with the HRs of participants with the unhealthiest lifestyle but in the least affluent SES group (i.e. unhealthy + high SES vs. unhealthy + low SES).<br><br>We will compare results for tests of interaction between lifestyle factor combinations and SES measures. |
| Outcomes | Primary outcome:<br>• All-cause mortality<br><br>Secondary outcomes:<br>• CVD and cancer mortality<br>• CVD and cancer incidence |
| Study | Prospective observational cohort studies.<br>Studies published in English language. |
| Exclusions | Ineligible publication/study design (e.g. reviews, conference abstracts, case control and cross-sectional studies, intervention studies, qualitative studies).<br><br>Studies lacking exposures or outcomes of interest (e.g. combinations of fewer than three lifestyle factor or SES not examined).<br><br>Studies that do not provide an assessment of the impact of SES on the association between combinations of lifestyle factors and adverse health. |

SES = socioeconomic status, CVD = cardiovascular disease.

## Population

This review will focus on the impact of SES on lifestyle associated adverse health outcomes in the general population. Of the studies included in previous systematic reviews investigating the adverse health outcomes associated with combinations of lifestyle factors, very few

For peer review only

included an evaluation of the impact of SES.(5, 8, 33) Therefore, because it was anticipated that few studies would fit the inclusion criteria, the population type was not restricted in order to identify as many studies as possible.

## Exposure

Only studies that examine both combinations of lifestyle factors and SES as exposure variables will be included. Studies that examine the combined influence of at least three lifestyle factors will be included. It was decided that three lifestyle factors represented a balance between identifying the evidence for combinations of lifestyle factors as opposed to single lifestyle factors (two lifestyle factors was felt to be too narrow) whilst ensuring that a sufficient number of studies are included (there are fewer studies examining the risks of >3 lifestyle factors). In order to help identify as much literature as possible we decided that all definitions of SES variables will be accepted.

## Comparator

Studies will be included if they examine the effect of SES on the associations between combinations of lifestyle factors and adverse health outcomes. Results for effects may be reported in different ways: hazard ratios, odds ratios, incidence rates. Where possible, comparisons of effects for similar outcomes will be made across studies. For example, the hazard ratios (HRs) of participants with the least healthy lifestyle factor combination in the most affluent SES group will be compared with the HRs of participants with the least healthy lifestyle combination but in the least affluent SES group. Where reported, we will compare results for tests of interaction between lifestyle combinations and SES measures.



For peer review only - http://bmjopen.bmj.com/site/about/guidelines.xhtml

## Outcomes

Our primary outcome of interest is all-cause mortality. However, lifestyle-associated adverse health is well recognised to be strongly linked to CVD and cancer outcomes. Therefore, we will include studies that examine the following outcomes: CVD and cancer mortality; CVD and cancer incidence. Studies examining specific CVD or cancer outcomes such as stroke, angina, or site-specific cancer will also be included. The International Classification of Diseases (10$^{th}$ revision) codes I05–I89.9 and C00-C97 will be used to define CVD and cancer outcomes, respectively.

## Study Design

We aim to identify prospective observational cohort studies. Case-control and cross-sectional studies, intervention studies, qualitative work, and review articles will be excluded. Only full-text published articles will be included and conference abstracts, dissertations, editorials, or papers without data will be excluded. Studies not published in the English language will be excluded.

## Study Identification

### Electronic Searches

A systematic search of PubMed, EMBASE and CINAHL databases will be performed. The search strategy will incorporate a combination of Medical Subject Heading (MeSH) terms and keywords. The search strategy of a recent systematic review examining combined lifestyle factors and the risk of incident type 2 diabetes was used as a template and adapted to incorporate SES related MeSH terms and keywords.(8) The search strategy has been developed with assistance from a specialist university librarian. Table 2 shows the search



For peer review only - http://bmjopen.bmj.com/site/about/guidelines.xhtml

strategy that will be used for PubMed. The search strategy will be adapted and applied to other databases and will be available from PROSPERO once the review is complete.

## Table 2. PubMed search strategy

| Search | MeSH Terms and keywords | Theme |
|---|---|---|
| #1 | combination*[tiab] OR combined[tiab] OR composite[tiab] OR integrated[tiab] OR interaction*[tiab] OR joint effect* OR merged effect*[tiab] OR score*[tiab] OR adhere* to[tiab] OR collective[tiab] OR cumulative[tiab] OR multiple[tiab] | combined |
| #2 | life style[MeSH] OR life style*[tiab] OR lifestyle*[tiab] OR risk reduction behavior[MeSH] OR risk reduction behaviour*[tiab] OR health behavior[MeSH] OR health behaviour[tiab] OR health factor*[tiab] OR low risk*[tiab] OR prevention guideline*[tiab] OR protective factor*[tiab] OR risk reduction behaviour*[tiab] OR health* behaviour*[tiab] OR risk behaviour*[tiab] OR modifiable factors[tiab] | lifestyle factors |
| #3 | healthcare disparities[MeSH] OR healthcare disparities[tiab] OR Health Status Disparities[MeSH] OR disparate[tiab] OR disparit*[tiab] OR inequal*[tiab] OR health inequalities[tiab] OR unequal[tiab] OR health inequities[tiab] OR inequit*[tiab] OR socioeconomic factors[MeSH] OR socioeconomic factors[tiab] OR socio-economic*[tiab] OR socioeconomic*[tiab] OR social-economic[tiab] OR Social Determinants of Health[MeSH] OR social determinant*[tiab] OR poverty[MeSH] OR poverty[tiab] OR depriv*[tiab] OR sociological factors[MeSH] OR sociological factors[tiab] OR social medicine[MeSH] OR social medicine[tiab] | SES |
| #4 | cohort studies[MeSH] OR cohort[tiab] OR incidence[MeSH] OR incidence[tiab] OR survival analysis[MeSH] OR survival[tiab] OR early diagnosis[MeSH] OR early diagnosis[tiab] OR prospective*[tiab] OR follow* up[tiab] OR longitudinal[tiab] OR nested case-control[tiab] OR nested case control[tiab] OR predict*[tiab] | study design |
| #5 | #1 AND #2 AND #3 AND #4 | - |
| #6 | Death[MeSH] OR death*[tiab] OR mortality[MeSH] OR mortalit*[tiab] OR fatal*[tiab] OR life expectanc*[tiab] OR surviv*[tiab] | mortality outcome |
| #7 | cardiovascular diseases[MeSH] OR cardiovascular[tiab] OR CVD[tiab] OR heart disease*[tiab] OR myocardial ischaemia[tiab] OR AMI[tiab] OR IHD[tiab] OR CHD[tiab] OR coronary artery disease*[tiab] OR CAD[tiab] OR myocardial infarction[tiab] OR heart infarction[tiab] OR acute coronary syndrome[tiab] OR ACS[tiab] OR heart failure[tiab] OR sudden cardiac death[tiab] OR cerebrovascular disorder*[tiab] OR cerebrovascular accident*[tiab] OR cerebrovascular attack*[tiab] OR CVA[tiab] OR cerebrovascular disease*[tiab] OR CBVD[tiab] OR cerebral arterial disease*[tiab] OR stroke*[tiab] OR apoplex*[tiab] | CVD outcomes |
| #8 | neoplasms[MeSH] OR neoplas*[tiab] OR cancer*[tiab] OR carcinoma*[tiab] OR tumour*[tiab] OR malignanc*[tiab] | cancer outcomes |
| #9 | #6 OR #7 OR #8 | - |
| #10 | #9 AND #5 (final search) | - |

*[MeSH] = Medical Subject Heading; [tiab] = contained in either title or abstract; underlined = both UK and American spellings will be searched; *= any group of letters/characters, including no character.*

Searches will be from database inception (PubMed 1966; EMBASE 1947; CINAHL 1984) to 3rd March 2020. Searches will be supplemented by hand searching of reference lists of included papers, forward citation searching and a search for grey literature using the following sources:



For peer review only - http://bmjopen.bmj.com/site/about/guidelines.xhtml

- Charities/Health organisations: e.g. The King's fund, The Health Foundation, Cancer Research UK, World Health Organisation, American Heart Association, American Cancer Society
- Databases such as OpenGrey, the Healthcare Management Information Consortium, the National Technical Information Service
- Google and Google Scholar
- Literature compiled by governmental organisations e.g. Department of Health in England, Office for National Statistics, Centers for Disease Control and Prevention

## Data Collection and Analysis

### Study Selection

Studies identified by the search strategy will be uploaded to 'DistillerSR' software and duplicates will be removed. Two reviewers will independently screen titles and abstracts using the inclusion criteria above. Any conflicts shall be resolved by discussion and if an agreement cannot be made the study shall be included for full-text screening.

Full-texts will be reviewed using a piloted checklist based on the inclusion and exclusion criteria. Conflicts will be resolved by discussion and will include a third reviewer if no consensus is reached. All studies excluded at the full-text stage will be listed with reasons for exclusion given.



For peer review only - http://bmjopen.bmj.com/site/about/guidelines.xhtml

## Data Extraction

Each study that meets inclusion criteria after full-text screening will go through the data extraction phase. Data extraction will be carried out by two reviewers working independently using a piloted data extraction form (Box 1). Data will be extracted for the following study characteristics: author, publication year, title, study cohort, number of participants, proportion female, mean age, ethnicity, setting, country, date of recruitment, duration/follow up. Details of the lifestyle or metabolic factors and SES measures used as exposure variables will be extracted. Where possible, this detail will include how and when exposure variables were measured or assessed. Data will be extracted for any included metabolic factors such as BMI, blood pressure, or cholesterol levels. The number of study participants with unhealthy lifestyle factors will be recorded and reported. Details of confounder variables and the level of missing data will be extracted. Health outcome definitions and ascertainment will be recorded. The type of analysis, statistical methodology, and confounder adjustment will be extracted. Study results, the nature of adverse health outcome associations identified, and the effect sizes that measure the impact of SES on lifestyle associated outcomes will be recorded. Whether and how sensitivity analyses were conducted will be noted. Techniques for dealing with missing data will be recorded. Studies' overall conclusions will be extracted.

For peer review only



For peer review only - http://bmjopen.bmj.com/site/about/guidelines.xhtml

### Box 1. Data extraction

#### Article Identifiers

- Author
- Publication year
- Title
- Journal, Vol, Issue, Page numbers
- Source (e.g. Database, Grey Literature source, handsearching of references etc.)
- Study funding

#### Study characteristics

- Study aims and objectives
- Cohort name
- Number of participants
- Proportion female
- Mean age (SD)
- Ethnicity
- Country
- Setting (e.g. general population, occupational cohort etc.)
- Study duration/follow up
- Study start and end dates

#### Exposures, confounders, and outcomes

- Lifestyle and/or metabolic factors (definition; when and how measured/assessed)
- SES measures (definition; when and how measured/assessed)
- SES measures (definition; when and how measured/assessed)
- Confounder variables or covariates included
- Outcome definition
- Outcome ascertainment
- Number of participants with missing data

#### Analysis characteristics

- Type of analysis (statistical methods)
- Sensitivity analysis conducted
- Methods to deal with missing data

#### Results, conclusions, and quality

- Effect of SES on lifestyle-associated adverse health: yes or no
- Size of effect
- Study conclusions
- NOS score and justification

## Quality

Included studies will be assessed for quality using an adapted version of the Newcastle-Ottawa Scale for cohort studies (NOS),(34) a tool that has been used extensively for the appraisal of observational studies as described here. This scale, after piloting, has been adapted to include an assessment of confounder adjustment, sensitivity analyses, and dealing with missing data (Box 2).



For peer review only - http://bmjopen.bmj.com/site/about/guidelines.xhtml

## Box 2. Adapted Newcastle-Ottawa Scale †

### Selection

1) Representativeness of the cohort
   - a) truly representative of those about whom the study makes conclusions (adult/general/low income population) *
   - b) somewhat representative *
   - c) selected group of users e.g. nurses, volunteers
   - d) no description of the derivation of the cohort

2) Ascertainment of exposures (lifestyle factors and SES measures)
   - a) secure record (e.g. health care records) *
   - b) structured interview *
   - c) written self-report
   - d) no description

3) Analysis rigour
   - a) impact of >1 socioeconomic measure assessed*
   - b) impact of 1 socioeconomic measure assessed

4) Demonstration that participants were disease free at start of study
   - a) yes/sensitivity analysis excluding those with prior disease *
   - b) no

### Comparability (2 stars available)

1) Comparability of exposed vs unexposed on the basis of the design or analysis
   - a) analysis includes adjustment for key variables and maximises the use of available data*
   - b) study offers reasonable justification for adjustment variables *

### Outcome

1) Assessment of outcome
   - a) independent blind assessment *
   - b) record linkage *
   - c) self-report
   - d) no description

2) Was follow-up long enough for outcomes to occur
   - a) yes (≥6months) *
   - b) no

3) Adequacy of follow up of cohorts
   - a) all participants accounted for and missingness <50%, or assessment of impact of missingness given, or description of those with missing data allows assessment of impact of missingness*
   - b) some participants unaccounted for or missingness >50%, or no assessment of [illegible]

†A study can be awarded a maximum of one star for each numbered item within the Selection and Outcome categories. A maximum of two stars can be given for Comparability. A total of 9 stars are available: selection (4 stars), comparability (2 stars), and outcome (3 stars). If a single study combines/harmonises multiple cohorts for analysis, then the item below that best fits with the majority of the cohorts included should be given for that study. As per original NOS, for Selection, criterion 4) Demonstration that participants were disease free at start of study: 'In the case of mortality studies, outcome of interest is still the presence of a disease/ incident, rather than death. That is to say that a statement of no history of disease or incident earns a star.'(34)

## Data Synthesis

The process and results of study identification and selection based on inclusion and exclusion criteria will be displayed as a Preferred Reporting Items for Systematic Reviews and Meta-Analyses (PRISMA) flow diagram.(35)

To aid comparisons across studies we will present at least the following summary data for each included study in tabular format:

- Cohort characteristics (e.g. number of participants, proportion female, mean age, setting, date of recruitment, length of follow up)
- Lifestyle or metabolic factors included in combination
- SES measures used in assessment of SES effects
- Health outcomes and outcome ascertainment
- Risks for health outcomes and their statistical significance
- Inconsistent findings within each study
- Study quality



For peer review only - http://bmjopen.bmj.com/site/about/guidelines.xhtml

Studies will be grouped together according to our outcomes of interest (all-cause mortality; CVD and cancer mortality; CVD and cancer incidence). For all outcomes, we will describe details of how the outcome was assessed (e.g. administrative data or questionnaires) and approach used for analysis (e.g. time-to-event). We will provide details of the association between SES, lifestyle and the outcome (e.g. odds ratio, hazard ratio, etc) including the length of follow up. We will report studies attempts to deal with confounding and discuss whether resulting associations are likely confounded. Where possible, comparisons of effects for similar outcomes will be made across studies. For example, we will compare HRs of participants in the least healthy category and most affluent SES category (reference group) with HRs of participants in the least healthy category but in the most deprived SES category.

If studies are sufficiently homogenous in terms of participant characteristics, exposures, and outcomes, we will standardise study findings for similar outcomes, provide justification for our transformation methods, and combine results by a random-effects meta-analysis.(36) We will then calculate $I^2$ to describe the proportion of effect estimate variance due to study heterogeneity rather than chance.

Initial scoping of the literature has identified significant exposure, outcome, and methodological heterogeneity across studies. Therefore, a synthesis without meta-analysis (SWiM) will likely be the most appropriate method to synthesise study findings.(37) As per SWiM, we will provide justifications for the method and presentation of study findings.



For peer review only - http://bmjopen.bmj.com/site/about/guidelines.xhtml

Irrespective of whether a meta-analysis is conducted we will provide a transparent and full account of any limitations of our synthesis. Further, in conducting and reporting this systematic review we will endeavour to fulfil, where possible, all items proposed by the Meta-analysis Of Observational Studies in Epidemiology (MOOSE) Group.(38) Any amendments to the review protocol will be identified and justified upon completion.

## Patient and Public Involvement

This Systematic Review constitutes a primary aspect of HF's doctoral thesis. NHS Research Scotland Primary Care Patient and Public Involvement (NRS PPI) Group was consulted twice as part of preparatory work for the doctoral thesis funding application.(39) The NRS PPI Group were not involved in the design of the study but have influenced how results of this review, as well as other aspects of the doctoral thesis, will be presented at two planned public engagement events over the course of the thesis.

## Ethics, dissemination

This review will not require ethical approval as it will not involve individual-level patient data. Results will be disseminated via peer-reviewed publication, professional networks, social media, public events and conference presentations.

## Discussion

Both combinations of multiple unhealthy lifestyle factors and SES play major roles in mortality, CVD, and cancer.(1, 5, 8, 9, 22) Numerous studies have investigated the mediating influence of lifestyle factors in attempts to explain the socioeconomic gradient in adverse



For peer review only - http://bmjopen.bmj.com/site/about/guidelines.xhtml

health outcomes.(3, 4) However, fewer studies appear to examine the relationships between lifestyle, SES, and adverse health outcomes from the perspective of interactions between combinations of lifestyle factors and SES.(28) Understanding the evidence for whether and how SES influences the association between lifestyle and adverse health outcomes could inform policies and interventions that aim to support healthy lifestyles.

Scoping the literature suggests that evidence for a moderating influence of SES is mixed. Eguchi et al., with data from 42,647 Japanese adults aged 40–79 years and approximately 20 years follow-up, examined the risks associated with a lifestyle score (comprised of eight lifestyle factors: smoking, alcohol, physical activity, sleep, dietary intake of fruit, fish and milk, and BMI) stratified by age (≥16 or <16 years) at last formal education.(28) The authors reported a 44% higher all-cause mortality risk for those with a higher level of education but with the least healthy lifestyle, compared with those with a higher level of education but with the healthiest lifestyle. When the same comparison was made in those with the lower level of education, participants with the least healthy lifestyle had a 40% higher all-cause mortality risk compared with those with the healthiest lifestyle. Namely, the level of elevated risk associated with the least healthy lifestyle was similar in both higher and lower education groups.

Foster et al., performed similar analyses with data from 328,594 UK adults 40–69 years and approximately 5 years follow-up to examine the risks associated with a lifestyle score (comprised of nine lifestyle factors: smoking, alcohol, physical activity, television viewing time, sleep duration, and dietary intake of fruit/vegetables, oily fish, and red and processed meat) stratified by quintiles of socioeconomic deprivation (Townsend index).(6) The authors observed a 65% higher all-cause mortality risk for the least deprived with the least healthy

For peer review only



For peer review only - http://bmjopen.bmj.com/site/about/guidelines.xhtml

lifestyle, compared to the least deprived but with the healthiest lifestyle. However, when the same comparison was made in the most deprived participants, those with the least healthy lifestyle had a 145% higher risk than those with the healthiest lifestyles.

These highly comparable studies report opposing results. Eguchi et al. found no interaction between lifestyle and SES with similar lifestyle risks in the least and most educated groups. Whereas Foster et al. found an interaction between lifestyle and SES with disproportionately raised lifestyle risk in the most deprived group. Several methodological differences (lifestyle and SES measures; follow-up time; population characteristics) could explain the two studies' conflicting results but additional evidence from similar studies would help to clarify whether there is any moderating influence of SES on lifestyle-associated risks. This systematic review will help unpack such associations in more detail. In addition, included studies may identify specific combinations of unhealthy lifestyle factors that pose the highest risks for lower SES groups. However, we suspect there is likely to be a lack of studies which identify the combinations that pose the greatest risk for lower SES groups and this may be one of the evidence gaps that this review identifies.

To our knowledge, this systematic review will be the first to synthesise the evidence for whether and how SES influences the association between combinations of lifestyle factors and adverse health. We will describe the lifestyle factors, SES measures, and adverse health outcomes that have been examined thus far. We will collate and interpret the findings considering both the type of analyses and the quality of studies to provide a comprehensive synthesis of available evidence and highlight gaps in current evidence.



For peer review only - http://bmjopen.bmj.com/site/about/guidelines.xhtml

We have developed a comprehensive search strategy with broad inclusion criteria in order to identify all available evidence and reduce the chance of omitting relevant studies. However, our scoping of the literature suggests that not only will there be few studies that attempt to examine this problem but also that previous studies will be widely heterogeneous both in terms of the lifestyle factor and SES variables examined and in terms of the statistical methods employed. This will likely preclude a meta-analytical synthesis of the evidence, which may be a limitation of our review. However, a synthesis without meta-analysis (SWiM) will likely highlight important gaps in available evidence and direct future research in this sphere. This review will adhere to SWiM reporting guidelines and will be guided by MOOSE recommendations to improve transparency and clarity.(38, 40) All screening, data extraction, and quality assessment will be performed independently by two reviewers to improve study rigour.

This comprehensive and rigorous systematic review will improve our understanding of the complex interaction between socioeconomic status and lifestyle and has the potential to inform research, interventions, and policy.

## Author Contributions

HMEF, CAO'D, FSM and JMRG were involved in study concept. HMEF, CAO'D, FSM, PP and JMRG developed the study design. HMEF, CAO'D, FSM, JMRG and PP were involved in acquisition, analysis, or interpretation of data. Drafting of the manuscript was led by HMEF and PP with supervision and support from CAO'D, FSM and JMRG. All authors were involved



For peer review only - http://bmjopen.bmj.com/site/about/guidelines.xhtml

in critical revision of the manuscript for important intellectual content. CAO’D is the guarantor.

## Funding Statement

HF is funded by a Medical Research Council Clinical Research Training Fellowship (MRC CRTF; MR/T001585/1) and this body of work constitutes a primary aspect of HF’s doctoral thesis. The funder had no role in developing this protocol.

## Competing Interests Statement

The authors have no conflicts of interests to declare.

## Word Count

3,584 - excluding tables and boxes

# REFERENCES

1. Stanaway JD, Afshin A, Gakidou E, Lim SS, Abate D, Abate KH, et al. Global, regional, and national comparative risk assessment of 84 behavioural, environmental and occupational, and metabolic risks or clusters of risks for 195 countries and territories, 1990-2017: a systematic analysis for the Global Burden of Disease Study 2017. Lancet. 2018;392(10159):1923-94.
2. Meader N, King K, Moe-Byrne T, Wright K, Graham H, Petticrew M, et al. A systematic review on the clustering and co-occurrence of multiple risk behaviours. BMC Public Health. 2016;16(1).
3. Laine JE, Baltar VT, Stringini S, Gandini M, Chadeau-Hyam M, Kivimaki M, et al. Reducing socio-economic inequalities in all-cause mortality: a counterfactual mediation approach. International journal of epidemiology. 2019.
4. Marmot MG, Shipley MJ, Hemingway H, Head J, Brunner EJ. Biological and behavioural explanations of social inequalities in coronary heart disease: the Whitehall II study. Diabetologia. 2008;51(11):1980-8.
5. Loef M, Walach H. The combined effects of healthy lifestyle behaviors on all cause mortality: a systematic review and meta-analysis. Prev Med. 2012;55(3):163-70.
6. Foster HM, Celis-Morales CA, Nicholl BI, Petermann-Rocha F, Pell JP, Gill JM, et al. The effect of socioeconomic deprivation on the association between an extended

For peer review only

measurement of unhealthy lifestyle factors and health outcomes: a prospective analysis of the UK Biobank cohort. The Lancet Public Health. 2018;3(12):e576-e85.

7. Pepper GV, Nettle D. The behavioural constellation of deprivation: Causes and consequences. Behav Brain Sci. 2017;40:e314.

8. Zhang Y, Pan XF, Chen J, Xia L, Cao A, Zhang Y, et al. Combined lifestyle factors and risk of incident type 2 diabetes and prognosis among individuals with type 2 diabetes: a systematic review and meta-analysis of prospective cohort studies. Diabetologia. 2020;63(1):21-33.

9. Zhang YB, Pan XF, Chen J, Cao A, Zhang YG, Xia L, et al. Combined lifestyle factors, incident cancer, and cancer mortality: a systematic review and meta-analysis of prospective cohort studies. Br J Cancer. 2020;122(7):1085-93.

10. Jike M, Itani O, Watanabe N, Buysse DJ, Kaneita Y. Long sleep duration and health outcomes: A systematic review, meta-analysis and meta-regression. Sleep Med Rev. 2018;39:25-36.

11. Sun JW, Zhao LG, Yang Y, Ma X, Wang YY, Xiang YB. Association Between Television Viewing Time and All-Cause Mortality: A Meta-Analysis of Cohort Studies. Am J Epidemiol. 2015;182(11):908-16.

12. Krokstad S, Ding D, Grunseit AC, Sund ER, Holmen TL, Rangul V, et al. Multiple lifestyle behaviours and mortality, findings from a large population-based Norwegian cohort study - The HUNT Study. Bmc Public Health. 2017;17.

13. Verplaetse TL, McKee SA. An overview of alcohol and tobacco/nicotine interactions in the human laboratory. Am J Drug Alcohol Ab. 2017;43(2):186-96.

14. Linardakis M, Smpokos E, Papadaki A, Komninos ID, Tzanakis N, Philalithis A. Prevalence of multiple behavioral risk factors for chronic diseases in adults aged 50+, from eleven European countries - the SHARE study (2004). Prev Med. 2013;57(3):168-72.

15. Lawder R, Harding O, Stockton D, Fischbacher C, Brewster DH, Chalmers J, et al. Is the Scottish population living dangerously? Prevalence of multiple risk factors: the Scottish Health Survey 2003. Bmc Public Health. 2010;10.

16. Redondo-Bravo L, Fernandez-Alvira JM, Gorriz J, Mendiguren JM, Sanz J, Fernandez-Friera L, et al. Does Socioeconomic Status Influence the Risk of Subclinical Atherosclerosis? A Mediation Model. J Am Coll Cardiol. 2019;74(4):526-35.

17. Stringhini S, Zaninotto P, Kumari M, Kivimaki M, Lassale C, Batty GD. Socio-economic trajectories and cardiovascular disease mortality in older people: the English Longitudinal Study of Ageing. International Journal of Epidemiology. 2018;47(1):36-46.

18. Syden L, Landberg, J. The contribution of alcohol use and other lifestyle factors to socioeconomic differences in all-cause mortality in a Swedish cohort. Drug and Alcohol Review. 2017;36(5):691-700.

19. Groeniger JO, Kamphuis, C.B., Mackenbach, J.P., van Lenthe, F.J. . Repeatedly measured material and behavioral factors changed the explanation of socioeconomic inequalities in all-cause mortality. Journal of clinical epidemiology. 2017;91:137-45. Journal of Clinical Epidemiology. 2017;91:137-45.

20. Stringhini S, Sabia S, Shipley M, Brunner E, Nabi H, Kivimaki M, et al. Association of Socioeconomic Position With Health Behaviors and Mortality. Jama-J Am Med Assoc. 2010;303(12):1159-66.

21. Nordahl H, Lange T, Osler M, Diderichsen F, Andersen I, Prescott E, et al. Education and Cause-specific Mortality. 2014;25(3):389-96.

For peer review only



For peer review only - http://bmjopen.bmj.com/site/about/guidelines.xhtml

22. Stringhini S, Carmeli C, Jokela M. Socioeconomic status and the 25 x 25 risk factors as determinants of premature mortality: a multicohort study and meta-analysis of 1.7 million men and women (vol 389, pg 1229, 2017). Lancet. 2017;389(10075):1194-.

23. Katikireddi SV, Whitley E, Lewsey J, Gray L, Leyland AH. Socioeconomic status as an effect modifier of alcohol consumption and harm: analysis of linked cohort data. Lancet Public Health. 2017;2(6):E267-E76.

24. Pampel FC, Rogers RG. Socioeconomic status, smoking, and health: A test of competing theories of cumulative advantage. J Health Soc Behav. 2004;45(3):306-21.

25. Belsky DW, Caspi A, Cohen HJ, Kraus WE, Ramrakha S, Poulton R, et al. Impact of early personal-history characteristics on the Pace of Aging: implications for clinical trials of therapies to slow aging and extend healthspan. Aging Cell. 2017;16(4):644-51.

26. Fiorito G, Polidoro S, Dugue PA, Kivimaki M, Ponzi E, Matullo G, et al. Social adversity and epigenetic aging: a multi-cohort study on socioeconomic differences in peripheral blood DNA methylation. Sci Rep. 2017;7(1):16266.

27. Lewer D, Meier P, Beard E, Boniface S, Kaner E. Unravelling the alcohol harm paradox: a population-based study of social gradients across very heavy drinking thresholds. Bmc Public Health. 2016;16:599.

28. Eguchi E, Iso H, Honjo K, Yatsuya H, Tamakoshi A. No modifying effect of education level on the association between lifestyle behaviors and cardiovascular mortality: the Japan Collaborative Cohort Study. Sci Rep-Uk. 2017;7.

29. Andersen SW, Zheng W, Sonderman J, Shu XO, Matthews CE, Yu DX, et al. Combined Impact of Health Behaviors on Mortality in Low-Income Americans. Am J Prev Med. 2016;51(3):344-55.

30. Nordahl H, Lange T, Osler M, Diderichsen F, Andersen I, Prescott E, et al. Education and Cause-specific Mortality The Mediating Role of Differential Exposure and Vulnerability to Behavioral Risk Factors. Epidemiology. 2014;25(3):389-96.

31. Understanding the impact of socioeconomic status on the association between combined lifestyle factors and adverse health outcomes: a systematic review. PROSPERO 2020 CRD42020172588 [Internet]. 2020 [cited 25/06/2020]. Available from: https://www.crd.york.ac.uk/prospero/display_record.php?ID=CRD42020172588.

32. McKenzie J, Brennan, SE., Ryan, RE., Thomson, HJ., Johnston, RV., Thomas, J.,. Chapter 3: Defining the criteria for including studies and how they will be grouped for the synthesis. In: Cochrane Handbook for Systematic Reviews of Interventions version 6.0. . 2019.

33. Zhang YB, Pan XF, Chen J, Cao A, Zhang YG, Xia L, et al. Combined lifestyle factors, incident cancer, and cancer mortality: a systematic review and meta-analysis of prospective cohort studies. Br J Cancer. 2020.

34. Wells GA, Shea B, O'Connell D, Peterson J, Welch V, Losos M, et al. The Newcastle-Ottawa Scale (NOS) for assessing the quality of nonrandomised studies in meta-analyses: Ottawa Hospital Research Institute [Available from: http://www.ohri.ca/programs/clinical_epidemiology/oxford.asp.

35. Moher D, Liberati A, Tetzlaff J, Altman D. Preferred reporting items for systematic reviews and meta-analyses: the PRISMA statement [Guideline Research Support, Non-US Gov't]. Bmj. 2009;339:b2535.

36. Deeks J, Higgins, JPT., Altman, DG. . Chapter 10: Analysing data and undertaking meta-analyses. In: Cochrane Handbook for Systematic Reviews of Interventions version 6.0



For peer review only - http://bmjopen.bmj.com/site/about/guidelines.xhtml

(updated July 2019). Cochrane, 2019. Available from www.training.cochrane.org/handbook.; 2019.
37. Campbell M, McKenzie JE, Sowden A, Katikireddi SV, Brennan SE, Ellis S, et al. Synthesis without meta-analysis (SWiM) in systematic reviews: reporting guideline. BMJ. 2020;368:l6890.
38. Stroup DF, Berlin JA, Morton SC, Olkin I, Williamson GD, Rennie D, et al. Meta-analysis of observational studies in epidemiology: a proposal for reporting. Meta-analysis Of Observational Studies in Epidemiology (MOOSE) group. JAMA. 2000;283(15):2008-12.
39. The NRS Primary Care PPI Group [Available from: https://www.nhsresearchscotland.org.uk/research-areas/primary-care/get-involved.
40. Campbell M, McKenzie JE, Sowden A, Katikireddi SV, Brennan SE, Ellis S, et al. Synthesis without meta-analysis (SWiM) in systematic reviews: reporting guideline. Bmj-Brit Med J. 2020;368.

For peer review only



For peer review only - http://bmjopen.bmj.com/site/about/guidelines.xhtml

For peer review only

## PRISMA-P (Preferred Reporting Items for Systematic review and Meta-Analysis Protocols) 2015 checklist: recommended items to address in a systematic review protocol*

| Section and topic | Item No | Checklist item | Page number |
|---|---|---|---|
| **ADMINISTRATIVE INFORMATION** | | | |
| Title: | | | |
| Identification | 1a | Identify the report as a protocol of a systematic review | |
| Update | 1b | If the protocol is for an update of a previous systematic review, identify as such | 1 |
| Registration | 2 | If registered, provide the name of the registry (such as PROSPERO) and registration number | 3 |
| Authors: | | | |
| Contact | 3a | Provide name, institutional affiliation, e-mail address of all protocol authors; provide physical mailing address of corresponding author | 1 |
| Contributions | 3b | Describe contributions of protocol authors and identify the guarantor of the review | 1 |
| Amendments | 4 | If the protocol represents an amendment of a previously completed or published protocol, identify as such and list changes; otherwise, state plan for documenting important protocol amendments | 17 |
| Support: | | | |
| Sources | 5a | Indicate sources of financial or other support for the review | 21 |
| Sponsor | 5b | Provide name for the review funder and/or sponsor | 21 |
| Role of sponsor or funder | 5c | Describe roles of funder(s), sponsor(s), and/or institution(s), if any, in developing the protocol | 21 |
| **INTRODUCTION** | | | |
| Rationale | 6 | Describe the rationale for the review in the context of what is already known | 4-6 |
| Objectives | 7 | Provide an explicit statement of the question(s) the review will address with reference to participants, interventions, comparators, and outcomes (PICO) | 6 |
| **METHODS** | | | |
| Eligibility criteria | 8 | Specify the study characteristics (such as PICO, study design, setting, time frame) and report characteristics (such as years considered, language, publication status) to be used as criteria for eligibility for the review | 7-10 |
| Information sources | 9 | Describe all intended information sources (such as electronic databases, contact with study authors, trial registers or other grey literature sources) with planned dates of coverage | 11-12 |
| Search strategy | 10 | Present draft of search strategy to be used for at least one electronic database, including planned limits, such that it could be repeated | 11-12 (Table 2) |

| | | | |
|---|---|---|---|
| Study records: | | | |
| Data management | 11a | Describe the mechanism(s) that will be used to manage records and data throughout the review | 12 |
| Selection process | 11b | State the process that will be used for selecting studies (such as two independent reviewers) through each phase of the review (that is, screening, eligibility and inclusion in meta-analysis) | 12-13 |
| Data collection process | 11c | Describe planned method of extracting data from reports (such as piloting forms, done independently, in duplicate), any processes for obtaining and confirming data from investigators | 13-14 |
| Data items | 12 | List and define all variables for which data will be sought (such as PICO items, funding sources), any pre-planned data assumptions and simplifications | 13-15 |
| Outcomes and prioritization | 13 | List and define all outcomes for which data will be sought, including prioritization of main and additional outcomes, with rationale | 13-14 |
| Risk of bias in individual studies | 14 | Describe anticipated methods for assessing risk of bias of individual studies, including whether this will be done at the outcome or study level, or both; state how this information will be used in data synthesis | 14 |
| Data synthesis | 15a | Describe criteria under which study data will be quantitatively synthesised | 16-17 |
| | 15b | If data are appropriate for quantitative synthesis, describe planned summary measures, methods of handling data and methods of combining data from studies, including any planned exploration of consistency (such as $I^2$, Kendall's $\tau$) | 17 |
| | 15c | Describe any proposed additional analyses (such as sensitivity or subgroup analyses, meta-regression) | N/A |
| | 15d | If quantitative synthesis is not appropriate, describe the type of summary planned | 17 |
| Meta-bias(es) | 16 | Specify any planned assessment of meta-bias(es) (such as publication bias across studies, selective reporting within studies) | N/A |
| Confidence in cumulative evidence | 17 | Describe how the strength of the body of evidence will be assessed (such as GRADE) | 17 |

*** It is strongly recommended that this checklist be read in conjunction with the PRISMA-P Explanation and Elaboration (cite when available) for important clarification on the items. Amendments to a review protocol should be tracked and dated. The copyright for PRISMA-P (including checklist) is held by the PRISMA-P Group and is distributed under a Creative Commons Attribution Licence 4.0.**

*From: Shamseer L, Moher D, Clarke M, Ghersi D, Liberati A, Petticrew M, Shekelle P, Stewart L, PRISMA-P Group. Preferred reporting items for systematic review and meta-analysis protocols (PRISMA-P) 2015: elaboration and explanation. BMJ. 2015 Jan 2;349(jan02 1):g7647.*

For peer review only
